# The Association of Salivary Serotonin With Mood and Cardio-Autonomic Function: A Preliminary Report

**DOI:** 10.3389/fpsyt.2022.788153

**Published:** 2022-05-31

**Authors:** Michał Seweryn Karbownik, Steven Daniel Hicks

**Affiliations:** ^1^Department of Pharmacology and Toxicology, Medical University of Lodz, Łódź, Poland; ^2^Division of Academic General Pediatrics, Penn State College of Medicine, Hershey, PA, United States

**Keywords:** serotonin, salivary biomarkers, mood, depression, association

## Abstract

**Background:**

Serotonin plays an important role in mood regulation and depression. However, it is not clear whether the levels of serotonin in saliva are related to current mood.

**Aim:**

To test the association of salivary serotonin concentrations with mood, as well as cardiovascular and autonomic parameters.

**Materials and Methods:**

Saliva samples were obtained from collegiate runners and output parameters were examined before and after physical activity.

**Results:**

Salivary serotonin concentration was negatively associated with current mood (β = −0.32, 95%CI −0.62 to −0.02, *p* = 0.037, analysis adjusted for potential confounders), but insignificantly with measured cardiovascular and autonomic parameters.

**Conclusions:**

Salivary serotonin may reflect current mood. The results are preliminary and require further evaluation.

## Introduction

Serotonin (5-hydroxytryptamine, 5-HT) is a hormone and neurotransmitter of multiple biological functions. The majority of 5-HT is synthesized in the periphery; this happens in enterochromaffin cells of the gut and 5-HT is stored in the blood platelets. By contrast, only a small portion of bodily 5-HT functions in the central nervous system (CNS) (1), which represents a distinct pool of 5-HT (2, 3). Despite that, 5-HT in the CNS contributes to many neuropsychological processes (1, 4). Specifically, the link between central serotonergic neurotransmission and depression or mood regulation can be recognized in the depressogenic effect of 5-HT depleting agents such as reserpine, the antidepressant action of serotonergic medications, and the transient drops in mood resulting from tryptophan depletion in susceptible individuals (5). However, the serotonergic hypothesis of depression has not been well substantiated (5–8). Particularly, conflicting results have been reported regarding the association between 5-HT levels in peripheral body fluids and depressive symptoms or mood. For example, in relation to healthy controls, both decreased (9, 10) and increased (11) plasma 5-HT levels have been reported in depressed patients.

5-HT also appears to be involved in autonomic and cardiovascular function (12, 13). Peripheral 5-HT maintains blood vessel tone by vasoconstriction and endothelial-mediated vasodilatation (14), and is responsible for positive chronotropic and inotropic effects on the heart (15). In the CNS, the raphe nuclei serotonergic projections to autonomic brain areas mediate receptor-dependent tachy- or bradycardia as well as vascular tone regulation (12, 13). Previous human studies suggested plasma and platelet (16) as well as salivary 5-HT (17) to be positively linked to heart rate, but little attention has been paid on temporal changes within these parameters.

Among peripheral body fluids, saliva offers an attractive and easily accessible source of biomarkers with a potential for diagnostic and prognostic application in neuroscience (18, 19). 5-HT has been detected in saliva, being likely derived from the plasma (19, 20). In line, some studies attributed salivary 5-HT to peripheral indices (17, 21, 22), but no clear plasma-saliva relationship has been demonstrated for 5-HT (23, 24). On the other hand, although no solid evidence for the CNS origin of salivary 5-HT exists, saliva appears to reflect, to some extent, the CNS serotonergic status (23, 25, 26). It is not clear, however, whether salivary 5-HT measurement is relevant to mood assessment. Also, limited evidence exists regarding the relation of salivary 5-HT to autonomic or cardiovascular function (17).

The primary aim of the present study was to evaluate whether salivary 5-HT concentration is associated with current mood. Additionally, the study sought to examine the relationships between salivary 5-HT and several cardiovascular (blood pressure and heart rate) and autonomic parameters (body temperature and pupil diameter).

## Materials and Methods

### Study Design and Ethical Consideration

This was a secondary analysis of a dataset obtained from an observational cohort study employing a convenience sample of collegiate distance runners (27). The Independent Review Board at Marist College (Poughkeepsie, NY, USA) approved the study. Prior to enrolment, all the volunteers expressed their consent in a written form to participate in the study.

### Participants and Procedure

Collegiate distance runners of both sexes, aged 18-23, were approached during their weekly physical training. Runners suffering from acute illness or experiencing orthopedic injury in the previous week were excluded.

Before the run, all the participants self-reported some basal characteristics: socio-demographic (age, sex, ethnicity), physical parameters (height, body mass), substance use history (alcohol, nicotine, cannabinoid and opioid), depressive and anxiety symptoms over 2 weeks [assessed with Patient Health Questionnaire-9, PHQ-9 Scale (28) and Generalized Anxiety Disorder-7, GAD-7 Scale (29), respectively; all the participants regardless of depression/anxiety status were enrolled to the study], and oropharyngeal factors relevant to saliva collection (time since last tooth brushing, time since last meal, dietary restrictions) via written survey.

**Figure 1:**
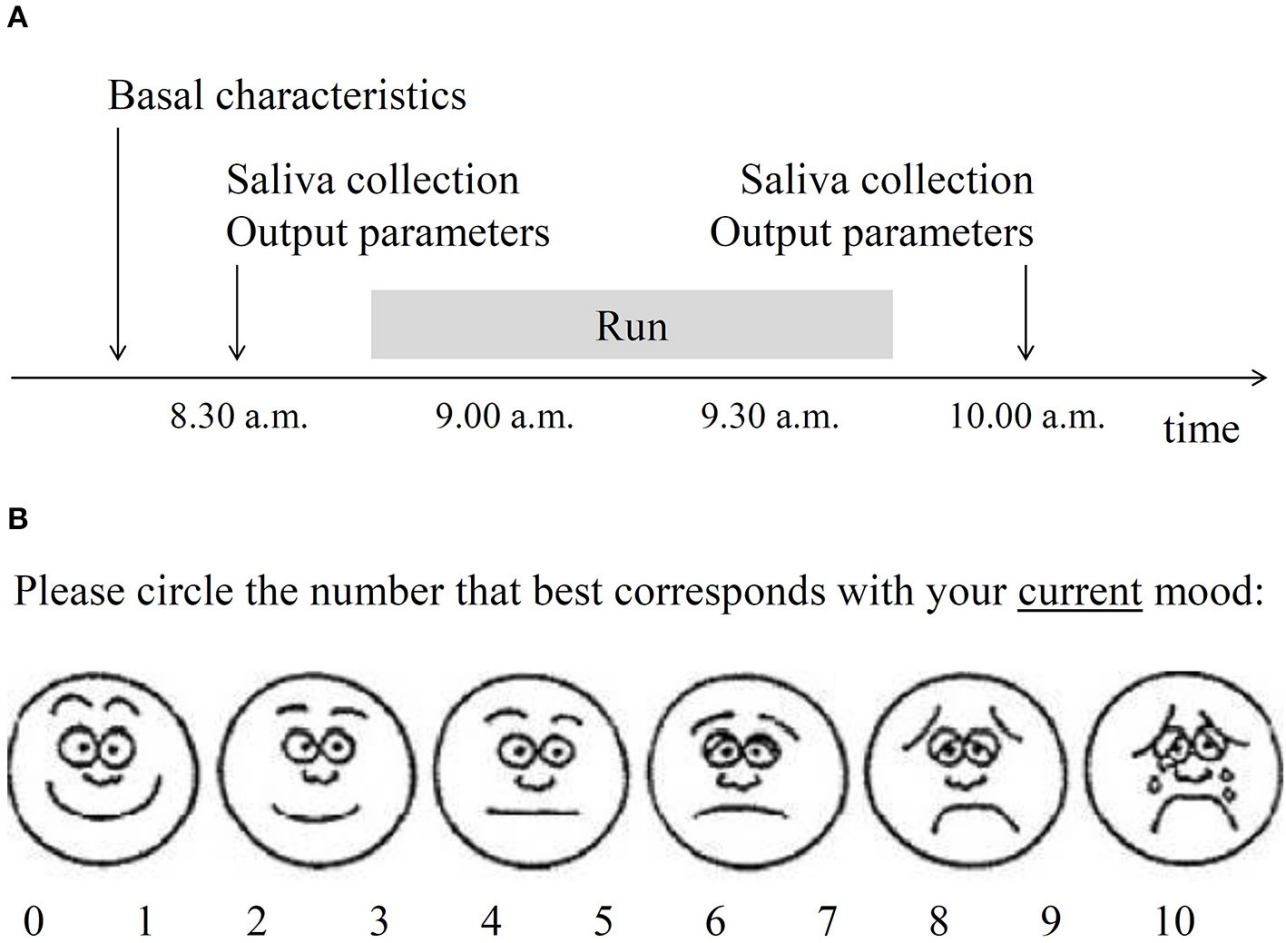
Selected methodological details of the study. **(A)** Timeline of the study. Basal characteristics included sociodemographic and physical parameters, substance use history, depressive, and anxiety symptoms over 2 weeks and oropharyngeal factors relevant to saliva collection. Saliva was collected to assess serotonin level. Output parameters included current mood **(B)**, blood pressure, heart rate, body temperature, and pupil diameter. **(B)** Visual analog mood scale used in the present study. The obtained score was reverse-coded to operationalize the construct of mood.

Saliva collection (to assess 5-HT concentration) and measurement of output parameters (mood, systolic and diastolic blood pressure, heart rate, body temperature and pupil diameter) were all performed at the same time twice: 10-15 min before the run (about 8.30 a.m.) and 10-15 min after the run (about 10.00 a.m.). The timeline of the study was presented in Figure 1A.

### Saliva Collection and Measurement of Output Parameters

Collection of whole saliva (at least 1 ml for a sample) was performed through active expectoration into 50 ml Falcon conical centrifuge tubes (Fisher Scientific; Waltham, MA, USA) following a water mouth rinse. The samples were kept on ice until they were transferred to be stored at −20°C until analysis.

Mood was assessed with the use of a pictogram-enhanced single-item 10-point visual analog mood scale (VAMS), illustrated by six facial expressions anchored between happy and sad. A participant was given a piece of paper with the VAMS and was asked to circle the number that best corresponded to her/his mood at that time (Figure 1B). Pictogram-enhanced visual analog scales are very brief tools that require minimum cognitive and linguistic involvement, produce negligible questionnaire burden, and enable respondents to express freely (30, 31); thus such tools appear suitable for sport research settings. Different formats of VAMS assessment have been successfully used in several other studies across age groups (32–34). Convergent validity of VAMS tools were confirmed against several standard mood assessing questionnaires and their test-retest reliability were satisfactory (30, 33, 35, 36).

Blood pressure was obtained in a seated position from the left arm using an adult-sized standard blood pressure cuff according to American Heart Association recommendations (37). Heart rate was measured by manually counting the radial pulse for 20 s. Pupil diameter was manually estimated by research staff using a standardized pupil diameter chart. Pupil diameter was measured indoors to ensure consistent ambient lighting conditions; participants were instructed to gaze directly at a blank wall 6 m away for 20 s before the examination to allow for pupillary accommodation. Body temperature was measured with a hospital-grade temporal artery thermometer (Exergen; Watertown, MA, USA) according to the manufacturer instructions. Excessive sweat was pat dry before post-run temperature assessment to avoid cooling resulted from evaporation.

### Salivary Serotonin Concentration Measurement

The technique of enzyme-linked immunosorbent assay (ELISA) was applied to determine 5-HT concentration in salivary samples. A ready-made kit Serotonin ELISA Fast Track (Rocky Mountain Diagnostics; Colorado Springs, CO, USA) was used according to the manufacturer instructions. The samples were thawed, centrifuged and supernatants were subjected to ELISA procedure in triplicates. The experimentally set intra- and inter-assay coefficients of variation were 10.5 and 13.2%, respectively, suggesting an acceptable technical performance for this assay.

### Data Analysis

As both salivary 5-HT and all output characteristics were measured twice: before and after the run (Figure 1A), pre- and post-run data for each participant was split into separate cases. As a result, there were twice as many cases as there were participants. This was done to increase statistical power of the analyses and was justified by different biological conditions of pre- and post-run measurements. To overcome a problem of inclusion of replicated cases, the analyses were confirmed using a bootstrap technique with 10,000 dataset resamples (38). General linear modeling was used to examine the association between salivary serotonin concentration and output parameters. As multiple hypotheses were tested, the Benjamini and Hochberg (B-H) procedure was used to control the false discovery rate at the level of 0.25 due to the exploratory nature of the analyses. *P*-values below the B-H corrected significance level were considered statistically significant. The analyses were performed using STATISTICA software version 13.3 (StatSoft; Tulsa, OK, USA).

## Results

### Characteristics of the Participants

Twenty-five athletes participated in the study. Their basal characteristics are presented in Table 1. The mean (± standard deviation) pre-run level of salivary 5-HT was 1,286 ± 873 ng/ml. Pre-run salivary 5-HT concentration was insignificantly related to socio-demographic data and physical characteristics (age *p* = 0.36, sex *p* = 0.20, body mass index *p* = 0.48).

**Table 1:** Basal characteristics of study participants.

**Characteristics**	**Mean (standard deviation) or absolute number (frequency)**
**Socio-demographics**	
Age [years]	20.0 (1.4)
Sex (females)	12 (48%)
Ethnicity (White)	23 (92%)
**Physical data**	
Body-mass index [kg × m^−2^]	20.4 (1.7)
**Substance use**	
Alcohol use in last 24 h	2 (8%)
Marijuana use in last 24 h	2 (8%)
**Mental health**	
Depressive symptoms (PHQ-9 score)	2 (0-5)^a^^b^
Anxiety symptoms (GAD-7 score)	3 (2–7)^a^^c^

*PHQ-9, Patient Health Questionnaire-9 Scale; GAD-7, Generalized Anxiety Disorder-7 Scale*.

a *Median (1^st^-3rd quartiles)*.

b *Four participants (16%) displayed mild depressive symptoms and three participants (12%) displayed moderate depressive symptoms*.

c *Seven participants (28%) displayed mild anxiety symptoms and three participants (12%) displayed moderate anxiety symptoms*.

### Association Between Salivary Serotonin and Output Characteristics

**Figure 2:**
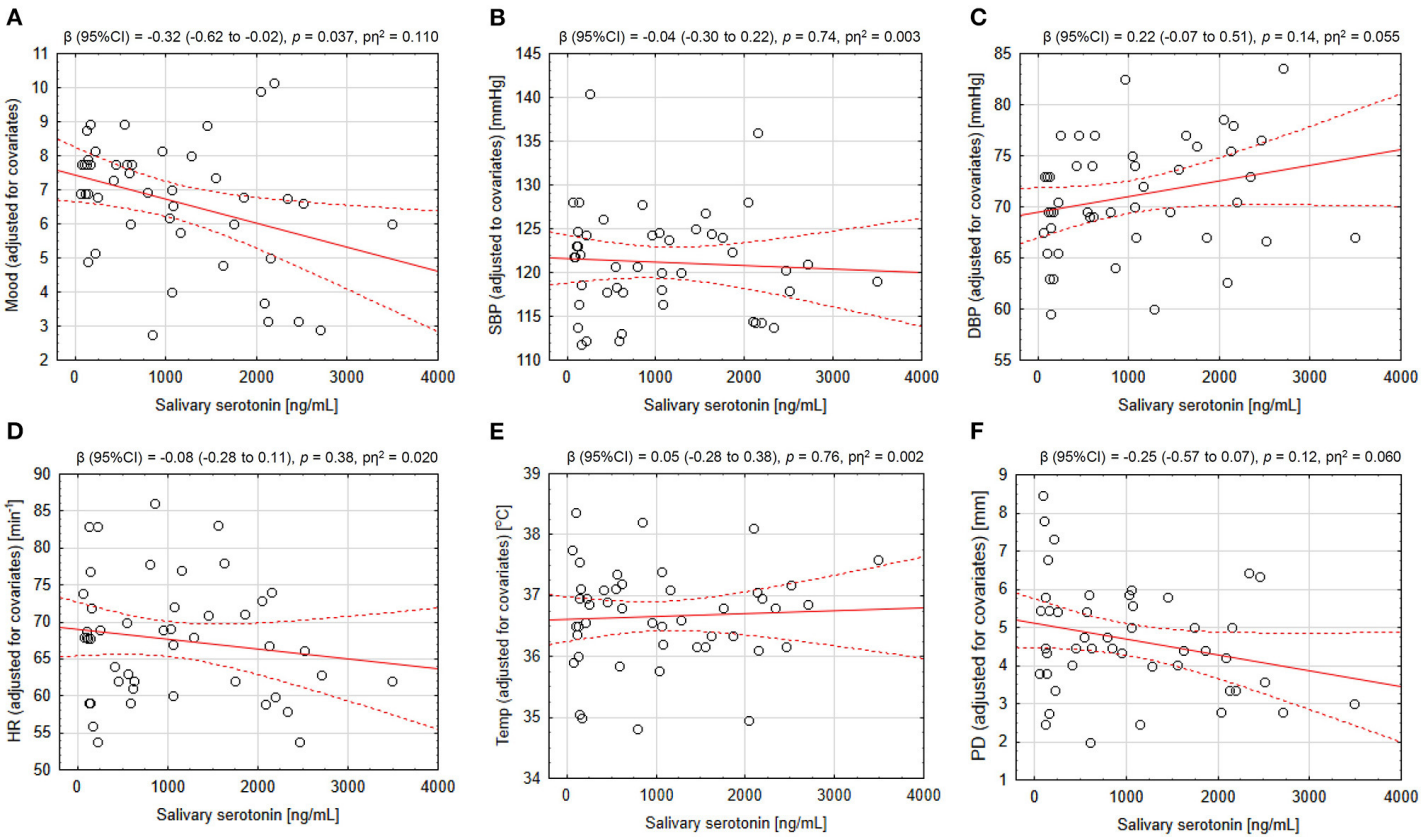
Correlation between salivary serotonin concentration and the output parameters in adjusted analyses. Individual data points are presented with best fitted least-squares regression lines and their 95% confidence intervals. Regression coefficients, *p*-values for the associations and effect sizes expressed as partial eta-squared values are presented above each scatter plot. **(A)** Mood, **(B)** systolic blood pressure, **(C)** diastolic blood pressure, **(D)** heart rate, **(E)** body temperature, and **(F)** pupil diameter. SBP, systolic blood pressure; DPB, diastolic blood pressure; HR, heart rate; Temp, body temperature; PD, pupil diameter; pη^2^, partial eta-squared.

The mood of athletes was found to be inversely correlated with their salivary 5-HT concentration and the extent of association was not affected by adjusting for potential confounders. The effect size of the association was similar in pre- as well as post-run settings, and there was no significant difference in the extent of association between sexes. The bootstrap analysis performed with 10,000 dataset resamples yielded similarly significant results. Heart rate was also linked to salivary 5-HT in a negative way, but adjusted analysis returned insignificant result. Other output characteristics were found not to be significantly related to salivary 5-HT. See detailed results in Table 2; Figure 2.

**Table 2:** Association between concentration of serotonin in saliva and output characteristics.

**Characteristics**	**β** **(95% CI)**, ***p*****-value**	
	**Raw analysis**	**Adjusted analysis^**a**^**
Mood^b^	−0.33 (−0.61 to −0.04), *p* = 0.025^c^	−0.32 (−0.62 to −0.02), *p* = 0.037^d^
Systolic blood pressure	−0.11 (−0.41 to 0.18), *p* = 0.44	−0.04 (−0.30 to 0.22), *p* = 0.74
Diastolic blood pressure	0.02 (−0.28 to 0.32), *p* = 0.90	0.22 (−0.07 to 0.51), *p* = 0.14
Heart rate	−0.29 (−0.58 to 0.01), *p* = 0.042^e^	−0.08 (−0.28 to 0.11), *p* = 0.38
Body temperature	0.12 (−0.17 to 0.41), *p* = 0.42	0.05 (−0.28 to 0.38), *p* = 0.76
Pupil diameter	−0.19 (−0.48 to 0.10), *p* = 0.20	−0.25 (−0.57 to 0.07), *p* = 0.12

*CI, confidence intervals*.

a *Adjusted for sex, time of testing (pre- or post-run), recent use of alcohol (2 = last 12 h, 1 = last 12-24 hours, 0 = no reported use or the use >24 h before sampling), recent use of marijuana (2 = last 12 h, 1 = last 12-24 h, 0 = no reported use or the use >24 h before sampling), no teeth brushing after last meal if it was eaten <2 h before sampling; smoking cigarette and using opioids was not included as covariate due to only one individual reporting using them; Benjamini and Hochberg corrected significance level was 0.042*.

b *Mood estimated with the visual analog mood scale was found inversely correlated with depressive and anxiety symptoms (measured by Patient Health Questionnaire-9 Scale and Generalized Anxiety Disorder-7 Scale, respectively) with borderline significance (r = −0.27, p = 0.058; r = −0.25, p = 0.082, respectively); pre-run salivary 5-HT was inversely but insignificantly related to depressive and anxiety symptoms (r = −0.24, p = 0.26; r = −0.20, p = 0.35, respectively)*.

c *Bootstrap analysis performed with 10,000 dataset resamples yielded similar result: β = −0.33, p=0.021; sex^×^serotonininteraction in predicting the mood was insignificant (p = 0.49); there was no significant sex difference in mood (p = 0.69)*.

d *Effect size of the adjusted association was similar in pre- and post-run settings (β = −0.25 pre-run and β = −0.26 post-run) as well as in the combined dataset; bootstrap analysis performed with 10,000 dataset resamples yielded similar result: β = −0.32, p = 0.035; sex^×^serotonin interaction in predicting the mood was insignificant (p = 0.70); there was no significant sex difference in mood (p = 0.86)*.

e *Effect size of the raw association was lower in pre- and post-run settings (β = −0.07 pre-run and β = −0.08 post-run) than in the combined dataset*.

## Discussion

The role of 5-HT in the pathogenesis and therapy of depressive disorders has been originally appreciated (5–8). However, the literature reports inconsistent results regarding the link between 5-HT in the periphery, including the saliva, and depression or mood (21, 26). Here, we show that salivary 5-HT may be inversely associated with an individual's current mood.

CNS and periphery constitute two distinct 5-HT pools (2, 3) and saliva appears to reflect peripheral more than central 5-HT content (17, 20–22, 24). Nevertheless, Matsunaga et al. (25) found salivary 5-HT to be linked with happiness in socially-positive settings. As happiness appears to be constituted by higher CNS functioning (39), this may support the hypothesis of salivary 5-HT relevance to CNS processes. Importantly, the association between happiness and salivary 5-HT level discovered by Matsunaga et al. (25) was inverse, which is in line with the present findings; yet happiness, positive mood or just subjective well-being are all similar and overlapping constructs (40–42). Moreover, Tan et al. (26) revealed that diurnal rhythm of salivary 5-HT concentration differs between healthy subjects and patients suffering from depression. In their report, healthy individuals tended to display lower morning levels of salivary 5-HT than depressed individuals before and after pharmacological treatment (26). This finding is consistent with the current study, which also relied on morning saliva samples. Additionally, following acute treatment of depressed individuals with a selective serotonin re-uptake inhibitor, a slight increase in plasma 5-HT (from which salivary 5-HT likely arises) has been reported (43), whereas long-term treatment of more than 4 weeks (time relevant to clinical improvement) leads to a decrease in plasma 5-HT (9, 44). This decrease is more pronounced in patients whose depressive symptoms ameliorate with treatment (9). Collectively, the current findings align with literature reports relevant to peripheral 5-HT.

The reported negative association between salivary 5-HT level and current mood might be somewhat specific to the athletes. It appears that increased extracellular brain 5-HT contributes to development of fatigue during prolonged exercise (45). In laboratory animals, serotonergic 5-HT_1A_ receptor stimulation diminished physical activity endurance, particularly in highly active animals (46). Therefore, the increase in salivary 5-HT may reflect accumulating fatigue during training, which may lead to lowered mood (47, 48). The midbrain 5-HT levels may adaptively increase following chronic endurance exercise (49). It will be interesting to see whether the negative relationship between salivary serotonin and mood, reported here, can be replicated in individuals with a sedentary lifestyle.

Although salivary 5-HT was found statistically significantly associated with mood, this measure is unlikely to serve as a stand-alone mood biomarker. To be such, a biomarker should be characterized by a substantial effect size in the link to the outcome as reflected by satisfactory discriminatory accuracy (19). Salivary 5-HT level in the present study could explain only 11% of variation in mood, leaving great majority potentially attributable to other factors. Biological processes are too much complex for a single molecular entity to accurately characterize an outcome. Instead, composite biomarkers may enable better predictions (50). Future studies should additionally investigate other salivary molecules potentially relevant to mental health (51, 52).

The reported negative association between salivary 5-HT and current mood was found to be stable irrespective of adjustment for confounding factors, particularly, post-exercise (27) and recent substance use (53), as these states may reduce salivary 5-HT and improve mood themselves. The effect size of the 5-HT-mood link in pre- and post-run states were similar to that observed in the combined dataset, which supports the veracity of the results. In comparison, heart rate, which was negatively linked to salivary serotonin in raw analysis, became insignificantly associated following adjustment for post-run status. Moreover, for heart rate, separate raw analyses in pre- and post-run states yielded much lower effect sizes than that of combined. On the other hand, despite insignificant raw analyses, diastolic blood pressure and pupil diameter presented some trends in adjusted analyses toward positive and negative association with salivary 5-HT, respectively. It is possible that cardiovascular and autonomic measures in the present study could be confounded by physical activity as the training and related increase in oxygen demand is well recognized to enhance these physiologic parameters (54). The existing literature suggests a positive link between peripheral 5-HT status and adrenergic functioning (16, 17, 55), and the use of serotonergic medications was associated with mydriasis (56), but no link was reported between peripheral 5-HT levels and blood pressure (16). Due to largely insignificant results in the present study, little coherence between raw and adjusted analyses and dissimilar literature reports, our results of cardiovascular and autonomic function may be regarded inconclusive.

The present study holds important limitations. First, it is a secondary analysis of existing data, which diminishes certainty of the obtained results (57). Second, the methods used are of limited validity; cardiovascular and autonomic functions were partially examined with subjective measures, however, the participants were accustomed to frequent manual heart rate assessment, as part of their training, and were likely to provide more accurate heart rate estimates than novice athletes. Mood was assessed with single-item pictogram-based VAMS questionnaire. Although the validity of such VAMS tools was proven (30, 33, 35, 36), rapid measuring may not be capable of distinguishing whether the assessed construct is more relevant to mood or emotion (34). On the other hand, as the assessment was related to the current state, it could track temporal changes secondary to possibly rapid fluctuation in salivary 5-HT (27, 58). Monitoring salivary 5-HT and mood “at the same moment” is a clear advantage of this study. Lack of association between depressive and anxiety symptoms with salivary 5-HT reported elsewhere (17), and only minimal and insignificant herein (PHQ-9 and GAD-7 scores), could be attributed to wide time-frame of assessed mood or not taking measurements at the same time. Third, saliva collection in this study was not preceded with sufficient instructions aimed at eliminating possible confounding effects of diet (59), brushing the teeth and other behaviors, as some other studies have done (17, 24–26); however, some relevant data was gathered to adjust the analysis for these confounders. Finally, the major barrier to generalization of the obtained results is high heterogeneity of reported salivary 5-HT concentration in humans, ranging from fractions of a nanogram per milliliter (21), through several dozens (17, 24–26), up to hundreds or even thousands of nanograms per milliliter (27, 60). High values of salivary 5-HT concentration are observed in the present study. This problem calls for validation of salivary 5-HT measurement techniques. Nevertheless, although these preliminary findings require reassessment in more valid research settings, the results add to a growing body of literature regarding the relevance of salivary 5-HT biomarker to neuroscience.

## Conclusions

Salivary 5-HT concentration appears negatively associated with current mood. The evidence of salivary 5-HT association with some cardiovascular and autonomic functions, such as blood pressure, heart rate, body temperature and pupil diameter measurement, is inconclusive. Although the result for salivary 5-HT and mood finds literature support, it is preliminary and requires further testing. Salivary 5-HT measures require standardization and validation before firm conclusions can be drawn.

## Data Availability Statement

The dataset for this study can be found in the Mendeley Data repository (http://dx.doi.org/10.17632/wkyrf5prjc.1).

## Ethics Statement

The study was reviewed and approved by Independent Review Board at Marist College. All the participants provided their written informed consent to participate in this study.

## Author Contributions

MK: conceptualization, formal analysis, visualization, and writing—original draft. MK and SH: data curation, funding acquisition, methodology, supervision, validation, and writing—review and editing. SH: investigation, project administration, and resources. Both authors have read and agreed to the published version of the manuscript.

## Funding

The research was supported by Quadrant Biosciences (Syracuse, NY, USA), Penn State College of Medicine (Hershey, PA, USA), Marist College (Poughkeepsie, NY, USA), and Medical University of Lodz (Łódz, Poland, grant number 503/5-108-03/503-51-001-19-00).

## Conflict of Interest

SH has served as a paid consultant and scientific advisory board member for Quadrant Biosciences. The remaining author declares that the research was conducted in the absence of any commercial or financial relationships that could be construed as a potential conflict of interest.

## Publisher's Note

All claims expressed in this article are solely those of the authors and do not necessarily represent those of their affiliated organizations, or those of the publisher, the editors and the reviewers. Any product that may be evaluated in this article, or claim that may be made by its manufacturer, is not guaranteed or endorsed by the publisher.
